# Zanadamu: An African hominin isotopic dataset

**DOI:** 10.1016/j.dib.2023.109522

**Published:** 2023-08-25

**Authors:** Victor Iminjili, Ricardo Fernandes

**Affiliations:** a Department of Archaeology, Max Planck Institute of Geoanthropology, Kahlaische Strasse 10, 07745, Jena, Germany; b Department of History, Geography and Communication, Universidad De Burgos, Paseo de Comendadores, s/n 09001, Burgos, Spain; c Climate Change and History Research Initiative, Princeton University, Princeton, USA; d Arne Faculty of Arts, Masaryk University, Nováka 1, 602 00 Brno-střed, Czechia

**Keywords:** Stable carbon isotopes, Stable oxygen isotopes, Tooth enamel, Hominins, Human evolution

## Abstract

The present article introduces Zanadamu, a comprehensive geo-temporal-referenced dataset that amalgamates all published stable isotope carbon and oxygen measurements on tooth enamel from African hominins, dated between 4.4 and 0.005 Ma. Zanadamu serves as a research tool for investigating hominin evolution by facilitating the examination of how different hominin species explored food resources and interacted with their local paleoenvironments. The dataset is structured in a machine-readable format, and its metadata organization allows for facile statistical analyses and comparisons with other types of isotopic records, including ancient and modern humans and other primates. Zanadamu is part of the AfriArch data initiative, which aims at compiling datasets for the study of ancient Africa. This an active initiative, and we strive to update Zanadamu as novel data becomes available.

Specifications Table

**Table:** 

Subject	Palaeontology (Earth and Planetary Sciences)
Specific subject area	Hominin stable carbon and oxygen isotope measurements on tooth enamel
Type of data	Table
How the data were acquired	Bibliographic research: data was retrieved from publications with stable carbon and oxygen isotope measurements on tooth enamel from different hominin species.
Data format	Secondary
Description of data collection	Data was located using the online scientific search engine Google Scholar. We located original publications and compilations containing stable carbon and oxygen data from African hominin species. From these we extracted metadata information concerning isotopic measurements, taxonomic descriptions, and the location and chronology of the samples.
Data source location	All sources of raw data are identified in the dataset.
Data accessibility	Repository name: Pandora Data identification number: https://www.doi.org/10.48493/r9pa-me14 Direct URL to data: https://pandoradata.earth/dataset/zanadamu

## 1 Value of the Data

• Zanadamu is a research tool for paleoanthropologists, archaeologists, modern and paleo- ecologists, and researchers undertaking isotopic research
• Stable carbon isotope data measured on tooth is used to investigate spatiotemporal dietary patterns of ancient African hominins
• Stable carbon and oxygen isotope data measured on tooth enamel is used to investigate local paleoenvironmental conditions
• Isotopic hominin data can be compared with other isotopic datasets (e.g., modern and ancient humans and primates) to investigate developments in dietary habits and ecological transformations. It offers also a reference for novel hominin isotopic measurement.

## 2 Objective

Information on past hominin exploitation of food resources and environmental interactions is necessary to better understand evolutionary processes [1,2]. Stable carbon and oxygen isotope measurements on the remains of tooth enamel from individual hominins provide direct evidence on their dietary habits and coeval paleoenvironmental conditions. Carbon stable isotope ratios (δ^13^C) inform on food sources and feeding habitat. Plants following the dominant C_3_ or C_4_ photosynthetic pathways have clearly distinct δ^13^C values. These can be influenced by various paleoenvironmental factors, such as forest cover thickness or aridity levels, which also determine predominant vegetation cover. Oxygen stable isotope ratios (δ^18^O) are used to infer past temperatures and precipitation patterns and link hominin evolution to climate change. There was a recent review of hominin carbon isotopic data although this excludes individuals not identified to species level [3]. Furthermore, until now there has been no compilation that combined together carbon and oxygen isotopic measurements in a single geo- and temporal-referenced dataset together with machine-readable descriptions of taxonomy, dating methods, ancient habitat descriptions, and full bibliographic references. The Zanadamu dataset fills this gap and offers a research tool to be used by paleoanthropologists, archaeologists, ecologists, and isotope researchers involved in the study of past and modern primates.

## 3 Data Description

Data entries associated to hominin specimens are internally identified within the dataset using a sequence of integer numbers given in the field Zanadamu_ID. In addition, catalogue/lab numbers as per original publication are given under the field Skeleton_ID. When multiple measurements are available for the same skeleton, they are labeled using the Replicates_ID field. Values matching in this field indicate that the replicates belong to the same skeleton. Multiple fields are employed for taxonomic description (Subfamily, Tribe, Subtribe, Genus, and Species). The name of the site where the skeleton was discovered using local place name (Site) plus the name of the country (Country) and region (Region) are also provided. Site Latitude (Latitude) and Longitude (Longitude) coordinates are given in decimal degrees relative to the WGS84 reference system. The categorical field Biome describes the reconstructed paleoenvironment associated to the hominin specimen using the reconstruction reported in the original source publication. The field C3_per_diet reports estimated percentage of C3 (versus C4) diet as a mean and standard deviation.

The field ‘Material’ describes the material on which the isotopic measurements were carried out. In our dataset these are all tooth enamel measurements but the field is included in the future possibility that other types of materials are included. The field Tooth_type is used to describe the type of tooth on which the isotopic measurements were carried out. Capital letters for tooth type denote upper/maxillary teeth while lower case letters denote lower/mandibular teeth. R/r stands for upper right/lower right and L/l for upper left/lower left. These letters are combined with letters that identify the type of tooth: I/i – Incisor, C/c – Canine, P/p – Premolar, M/m – Molar (e.g., RP4 represent the upper right pre-molar 4). Isotopic ratios for stable carbon (δ^13^C) and oxygen (δ^18^O) are reported as permille relative to the VPDB standard. The fields Min_Age and Max_Age report the chronological interval, in millions of years, associated to each individual. The three major measurement techniques employed to date the skeletal remains are given in the fields Dating_technique 1, Dating_technique 2, and Dating_technique 3. The dataset includes a field for bibliographic references (Reference) formatted in accordance to the guidelines of the American Journal of Biological Anthropology (AJBA) together with the unique digital identifier (DOI) of the publication.

**Fig. 1:**
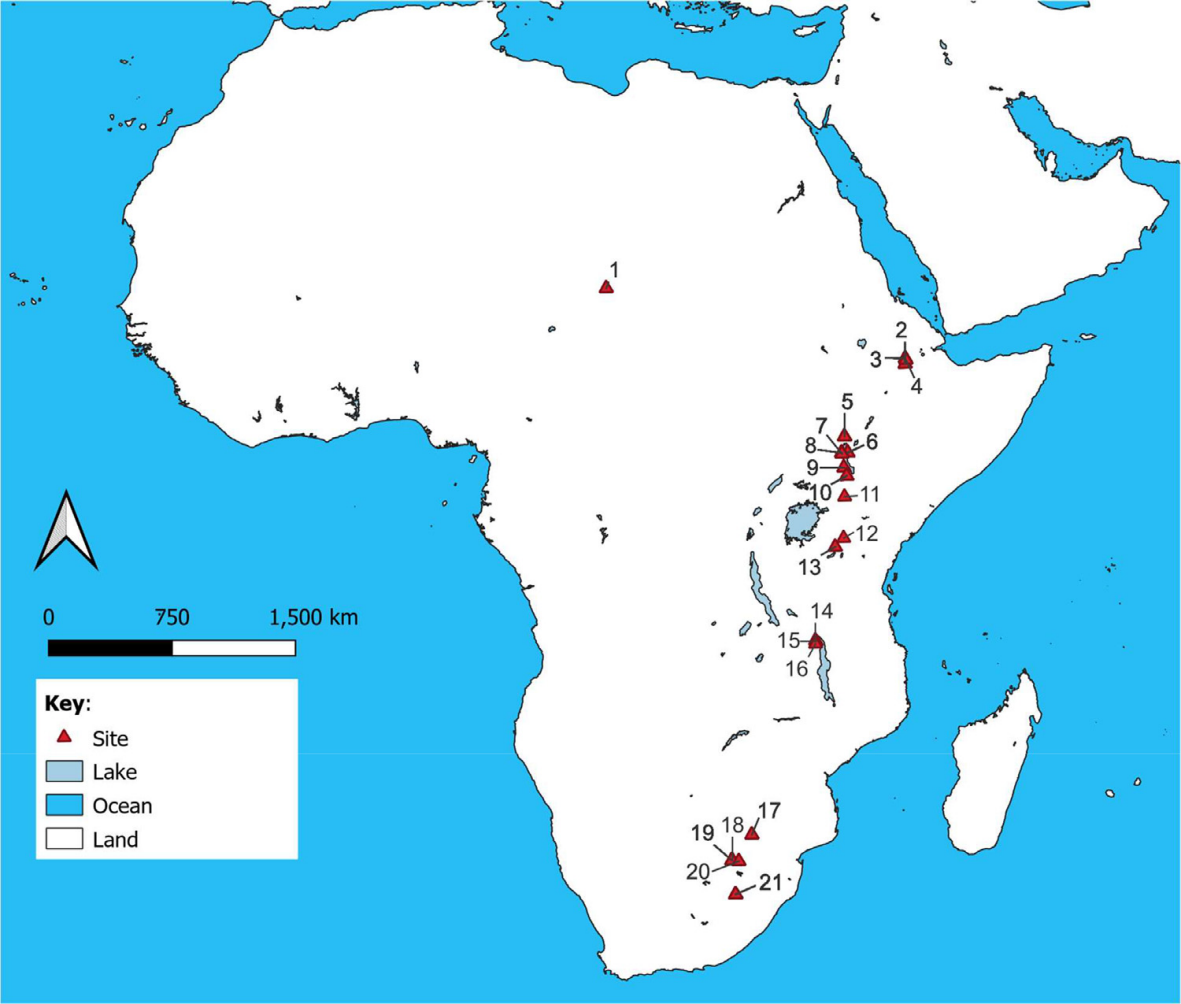
Spatial distribution of ancient hominin sites included in the Zanadamu dataset: (1) Koro Toro, (2) Aramis, (3) Hadar, (4) Dikika, (5) Lower Omo Valley, (6) Koobi Fora, (7) Lomekwi, (8) West Turkana, (9) Lothagam, (10) Kanapoi, (11) Baringo, (12) Peninj, (13) Olduvai Gorge, (14) Uraha-Karonga, (15) Mwenirondo-Karonga, (16) Malema-Karonga, (17) Makapansgat, (18 Kromdraai, (19) Sterkfontein, (20) Malapa, (21) Swartkrans.

Data was compiled from 16 publications [1,^2^,[4], [5], [6], [7], [8], [9], [10], [11], [12], [13], [14], [15], [16], [17]. It consists of individual 288 entries corresponding to 288 carbon isotopic measurements and 222 oxygen isotopic measurements from 21 African sites. The hominin species included in the dataset are listed in Table 1 and the spatial distribution of the site locations is shown in Fig. 1. The chronology for the samples ranges from 4.4 to 0.005 Ma. The data is made available online under Creative Commons Attribution-ShareAlike license at the AfriArch data community within the Pandora data platform. To the best of our knowledge, our data compilation includes all available stable carbon and oxygen isotopic measurements from African hominins.

**Table 1:** List of hominin species included in the Zanadamu dataset.

Species	Country	Geographic region	Min chronology (Ma)	Max chronology (Ma)
*Ardipithecus ramidus*	Ethiopia	Eastern Africa	4.4	4.4
*Australopithecus anamensis*	Kenya	Eastern Africa	4.12	3.95
*Australopithecus bahrelghazali*	Chad	Central Africa	3.58	3.58
*Australopithecus afarensis*	Ethiopia	Eastern Africa	3.5	3
*Kenyanthropus platyops*	Kenya	Eastern Africa	3.4	3
*Australopithecus africanus*	South Africa	Southern Africa	3	2
*Australopithecus sediba*	South Africa	Southern Africa	3	2
*Paranthropus aethiopicus*	Ethiopia Kenya	Eastern Africa	2.6	2.2
*Homo* *sp*	Ethiopia Kenya South Africa	Eastern Africa Southern Africa	2.55	1.46
*Homo rudolfensis*	Malawi	Southeastern Africa	2.5	2.3
*Paranthropus* boisei	Ethiopia Kenya Tanzania Malawi	Eastern Africa, Southeastern Africa	2.5	1.42
*Homo* *habilis*	Tanzania	Eastern Africa	1.8	1.75
*Paranthropus robustus*	South Africa	Southern Africa	1.8	1
*Homo sapiens*	Kenya	Eastern Africa	0.01	0.005

## 4 Experimental Design, Materials and Methods

To compile the hominin isotopic data, we undertook a bibliographic research using the scientific search engine Google Scholar using selected keywords such as “Africa”, “Hominin”, and “Isotopes”. We also relied on previous compilation of African hominin data [1], [2], [3],^9^,10]. In addition, we consulted non-stable isotope references to gather all necessary metadata. For instance, information on chronology and dating methods was often given in publications other than the stable isotope publication. The latitude and longitude coordinates for hominin sites were retrieved using the Google Maps web mapping platform. These may not correspond to the exact burial locations but we expect these to be located within a few kilometers of the location given in our dataset.

The dataset includes a field (C3_perc_diet) which reports estimates of dietary C3 (vs. C4) contributions for each specimen. To produce these, we employed the Bayesian mixing modelling software ReSources, an updated version of the software FRUITS [18,19]. Diet estimates relied on the enamel δ^13^C values for each specimen to which was associated a conservative uncertainty of 0.5‰. A mixing model with two end-members (C3 and C4 sources) was defined. Normally distributed δ^13^C values for these (C3: -11.2±2.3‰; C4: 3±1.1‰) are based on the modern δ^13^C vegetation compilation reported in Cerling et al. (1997) to which were added 14‰ to account for the diet-to-consumer enamel isotopic offset and 1.5‰ to account for atmospheric shifts in δ^13^C as a result of fossil-fuel burning [20]. The implemented model was deposited together with the dataset (file name: Zanadamu Bayesian mixing model.zip).

## Ethics Statements

This article meets the journal's Ethics and Policies requirements and does not involve animal or human studies.

## CRediT authorship contribution statement

**Victor Iminjili:** Conceptualization, Writing – original draft, Writing – review & editing, Data curation, Visualization, Validation. **Ricardo Fernandes:** Conceptualization, Writing – original draft, Writing – review & editing, Supervision, Formal analysis.

## Data Availability

Zanadamu (Reference data) (Pandora).
